# A Real-World Experience: Retrospective Review of Point-of-Care Ultrasound Utilization and Quality in Community Emergency Departments

**DOI:** 10.5811/westjem.58965

**Published:** 2023-06-30

**Authors:** Courtney M. Smalley, Erin L. Simon, McKinsey R. Muir, Baruch S. Fertel

**Affiliations:** * Cleveland Clinic Health System, Emergency Services Institute, Cleveland, Ohio; † Cleveland Clinic Lerner College of Medicine of Case Western Reserve University, Cleveland, Ohio; ‡ Cleveland Clinic Akron General, Department of Emergency Medicine, Akron, Ohio; § Northeast Ohio Medical University, Rootstown, Ohio; || Columbia University Vagelos College of Physicians and Surgeons, Department of Emergency Medicine, New York, New York

## Abstract

**Introduction:**

Point-of-care ultrasound (POCUS) is commonly used in the emergency department (ED) as a rapid diagnostic tool. Emergency medicine (EM) has been an early adopter of POCUS with indications expanding over the last 10 years. While the literature describes widespread use among academic sites, there is little data on clinical POCUS utilization at non-academic EDs. We sought to describe community emergency physician (EP) use of POCUS by quantifying the number and type of studies performed, characteristics of the performing physician, and quality metrics.

**Methods:**

Prior to the study period, all EPs underwent a standardized training and credentialing program. A retrospective review of all POCUS studies across 11 non-academic EDs from October 1, 2018–September 30, 2020 was performed by fellowship-trained physicians, who identified physician, exam type, and residency graduation year. The studies were then cross-referenced with quality review reports that assessed image acquisition, image interpretation, and image labeling. We performed descriptive statistics.

**Results:**

During the study period, 5,099 POCUS studies were performed by 170 EPs. Exams most frequently performed were cardiac (24%), focused assessment of sonography in trauma (21.7%), and pregnancy (16.2%). Recent EM residency graduates (<10 years) were higher utilizers of POCUS with a group mean of 1.3 exams per 100 patients. Of the studies done, 86% had no quality issues.

**Conclusion:**

Community POCUS demonstrates a heavy focus on core exams performed by recent EM residency graduates with minimal quality issues after a standardized training program. This study is the first to quantify actual community POCUS use in multiple EDs and may impact credentialing and skills maintenance requirements.

## INTRODUCTION

Point-of-care ultrasound (POCUS) use in bedside patient care is growing exponentially among multiple medical specialties and is a common modality used by emergency physicians (EP).^1^–^4^ In emergency medicine (EM), POCUS has been considered a valuable tool at the bedside since well before 2009, when the American College of Emergency Physicians (ACEP) adopted guidelines recommending formal residency training in POCUS.^1^,^5^,^6^ Trainees in EM are thus graduating with extensive experience in POCUS, and the frequency of use in clinical practice has grown.

While the literature describes the widespread use of POCUS among academic emergency departments (ED) and an expanding repertoire of novel indications, there is little data on clinical use among community EPs in locations without residency- or fellowship-training programs. In a 2011 study in which EM program directors were surveyed, researchers found that greater than half of graduating residents (57.1%) pursued careers directly in the community after graduation.^7^ Another study in 2019 confirmed this finding, reporting that 63% of residency graduates from a 10-year cohort accepted non-academic, community EM positions.^8^ Academic physicians are eager to expand POCUS. As it becomes more frequently used, it is essential to examine the use of this modality in community EM settings. In addition, it is important to determine current use of POCUS, the factors that affect its use, and potential quality issues since most EPs work in non-academic settings. This may help guide the implementation of training and quality programs for community POCUS and identify future training needs, credentialing, skills maintenance, infrastructure, and resources as POCUS expands across multiple specialties and among practitioners of various training levels.

For a variety of reasons POCUS has been enthusiastically adopted in academic settings, including the embracing of a novel technique, the presence of trainees, ED crowding, limited availability of radiology-based ultrasound or other imaging, and the ability to make a rapid bedside diagnosis. It is unclear whether a community setting with its associated demands on the clinicians, as well as potentially more resources, would engender widespread adoption of POCUS.

In our healthcare system, multiple community EDs have POCUS readily available to EPs. To standardize the POCUS program across multiple sites in the community and establish a reliable quality assurance (QA) program, a systemwide POCUS credentialing program was developed to assess the privileges of EPs between January 1, 2017–July 1, 2018. During this period, all academic, urban, suburban, and freestanding EDs were standardized in information technology workflow, machine purchasing, privileges, credentialing, coding/billing, and QA review.^9^ This allowed our physicians to move from ED to ED without any significant workflow or privileging changes and ensured the same level of quality across multiple departments (high reliability). A formal QA program was established at the end of this period.

In this study our objective was to review actual POCUS use among community EPs in a large healthcare system after implementing a standardized credentialing program. We assessed the types of studies performed, demographic data of physicians, number of years since residency graduation, number of studies performed per 100 patients, and quality metrics of POCUS studies. In addition, we aimed to determine the frequency of studies, the most-used exams, the effect of residency graduation year on use, and whether quality issues exist in the community, non-academic setting.

## METHODS

### Study Design

This retrospective, multicenter study involved 11 EDs across a large, integrated healthcare system in which all hospitals are located within one state. This study was implemented as part of a POCUS quality improvement project for the healthcare system and was institutional review board approved as exempt.

**Population Health Research Capsule**

What do we already know about this issue?

*Point-of-care ultrasound (POCUS) is commonly used in academic EDs as a diagnostic tool*.

What was the research question?

*What is the scope of POCUS use by community emergency physicians, by the quantified number and exam types, physician characteristics, and quality metrics?*

What was the major finding of the study?

*Most common exams performed were cardiac, FAST, and pregnancy. Recent residency completion was associated with higher POCUS use; 86% of studies had no quality issues*.

How does this improve population health?

*This study may help guide implementation of training and quality programs for community POCUS and identify future training needs, credentialing, and infrastructure*.

### Description of Credentialing Program Prior to Intervention Period

To obtain high quality data on community usage and quality measures, we performed the study after a standardized POCUS program was implemented across EDs in the system, as described in a previous paper.^9^ Briefly, this POCUS credentialing initiative was implemented from January 1, 201–July 1, 2018 across five urban community EDs, three suburban community EDs, and three freestanding EDs with a combined total of >500,000 patient encounters per year. Prior to the beginning of this program, all hospitals had different guidelines for POCUS privileges. To standardize POCUS privileges across the hospitals, a centralized credentialing process was created in which privileges were made identical across all hospitals. Uniform POCUS privileging was instituted based on current ACEP guidelines^6^ (Table 1). Each hospital’s medical executive committee approved privileges, thereby creating standardization across the healthcare system. Additionally, discrete electronic health record (EHR) orders, image archival workflow, templated documentation for study interpretation, and requirements for billing were identical across the hospitals. All hospitals were standardized to the same ultrasound machine, the Mindray TE-7 (Mindray North America, Mahwah, NJ) and the same EHR (Epic Systems Corporation, Verona, WI).

With regard to physician training, due to the large number of physicians a tier-based ultrasound credentialing system was employed such that physicians had to demonstrate competency in all exams within the tier to become credentialed for those studies. This was done to simplify the tracking of >150 EPs at 11 hospitals. To determine initial privilege groups, physicians were classified into two groups based on the date of residency graduation to determine a residency-based pathway group and a practice-based pathway group. Per previous publication, as recommended by the residency-based pathway of the ACEP policy statement, if a physician graduated during or after 2008, the physician was granted intermediate POCUS privileges.^9^ Physicians who graduated prior to 2008 were asked to provide a letter from their residency director, their residency program ultrasound director, or a supervisor at a previous job documenting previous POCUS training that met ACEP requirements.

The remainder of the physicians who did not meet the above criteria were required to attend an internal departmental POCUS course and undergo a practice-based pathway to obtain basic and/or intermediate credentialing. This practice-based POCUS credentialing pathway was supervised by the director of emergency ultrasound. This was a highly monitored program with details described in a prior paper.^9^ Additionally, to determine competency, physicians were allowed to schedule one-on-one scanning sessions with the director of emergency ultrasound if they had been actively scanning before the upgrades, and standardization made it possible for ultrasound leadership to track scans.

### Participants for Study

Any community EP working at an urban, suburban, or freestanding ED within the healthcare system who completed the ultrasound credentialing program described above was included in the study. We excluded physicians who primarily practiced at the main academic quaternary care hospital where an EM residency program was based and that also had POCUS fellowship-trained physicians on the faculty. If an EP worked at the academic site and a community site during the study period, the physician was included in the study and only their POCUS studies from the community sites were included in the dataset. In addition, any EP who joined the EM group practice after completion of the ultrasound credentialing program in 2018 was required to take the internal departmental POCUS course (the same course required for new physicians who underwent the practice-based credentialing pathway). This course was taught by EM POCUS leadership and reviewed the recommended image acquisition requirements for each POCUS exam: EHR orders, image archival, documentation, and chart requirements for billing.

### POCUS Workflow

A standard workflow was implemented across the system. Physicians placed an order in the EHR for the specific POCUS exam subtype desired: focused assessment of sonography in trauma (FAST); right upper quadrant for gallbladder pathology; pelvic for early pregnancy, etc. The order then generated a patient worklist on the POCUS machine within that individual ED. Those EDs with multiple machines used a shared worklist. The performing EP accessed the patient medical record for the exam and at the end of the exam, the images were pushed directly to the picture archiving and communicating system and to the EHR. No middleware was used. This image was immediately viewable and available for quality review to all EHR users across the system. In the EHR, the order created a reminder to interpret the image using an exam-specific and focused template recommended by the ACEP standardized reporting guidelines.

Each week, an automated report of all POCUS orders was generated and exported to a secure server. Information extracted include patient name, medical record number, date and time of study, type of study subtype as defined by the order placed in the EHR, and image interpretation of study. This database was used for QA by ED POCUS leadership.

Concurrently, a recommended POCUS image compendium was distributed to all EPs (Appendix 1). The compendium identifies standard views to be obtained as well as labeling nomenclature to be used when scanning. Additionally, each machine at all hospitals was loaded with standardized, preset descriptor labels to affix on the images.

### Intervention, Data Collection, and Quality Assurance Process

All POCUS studies performed by credentialed EPs from October 1, 2018–September 30, 2020 were included in the analysis. We excluded studies that were performed for educational purposes, performed by non-EPs (consultant), or non-credentialed EPs’ ultrasound exams. We conducted statistical analysis using SAS software (SAS Institute, Inc., Cary, NC). We computed descriptive statistics as well as medians and means.

Data extracted included the date of the POCUS exam, location, POCUS exam type, attending physician, physician interpretation, and QA findings. Additionally, each physician’s date of hire and residency graduation date was identified. The POCUS exam type was categorized by the EHR entry order. The POCUS exam was assigned to the attending physician who ordered the study through the EHR. For this study, we examined the number of residency cohort groups in five-year increments to determine how POCUS usage has changed over small increments of time.

The ED POCUS QA program required an ultrasound fellowship-trained EP to review all studies. All studies were reviewed within four weeks of acquisition. Each was reviewed for three focus areas: 1) image acquisition, defined as the “ability to acquire the required images for a particular POCUS study as defined by the image compendium”; 2) image interpretation, defined as “the ability for the physician to make the correct interpretation to answer the defined clinical question”; and 3) labeling of images, defined as “labeling of images such that independent reviewers of POCUS images can determine the anatomical location of the study.” When a reviewer doubted one of the qualitative quality measures, an additional POCUS reviewer was consulted.

A monthly QA report summarizing all exams and pertinent teaching points was sent to all EPs. As needed, specific feedback was sent to the performing physician, and cases were sent to peer review if a significant quality issue was identified.

## RESULTS

During the two-year study period, a total of 5,099 POCUS studies were performed across 11 community EDs within the healthcare system. A total of 170 EPs met inclusion criteria; 29 were excluded as they worked exclusively at the academic center. Table 2 demonstrates the number and percentage of the most common exams performed. In the community, limited cardiac, FAST, and limited pregnancy exams were the most common exams, accounting for 61.9% of total studies. The most infrequently performed exams in the community were deep venous thrombosis (DVT), appendix, and testicular ultrasound.

Of the 170 community EPs performing POCUS, years of residency graduation were recorded as a determinant of exposure to POCUS. As demonstrated in Table 3, more recent EM residency graduates were higher utilizers of POCUS with a group mean of 1.3 exams per 100 patients. Residency graduates in EM from 2005–2009 and 2010–2014 had 0.83 exams per 100 patients and 0.80 exams per 100 patients, respectively. There was a significant decrease in exams per 100 patients for the 2000–2004 graduates. In the pre-2000 residency group, the mean number of POCUS exams per 100 patients was 0.78. However, when examining this physician group, two physicians who worked at trauma hospitals performed 85% (900/1,062) of exams in this category, which skewed the results. When we excluded these two physicians from the dataset, as they were thought to be outliers, the mean number of POCUS exam per 100 patients was 0.26.

When reviewing the QA data, we found that 4,395/5,099 (86%) of POCUS studies had no quality issues (86%) (Table 4). With regard to the specific quality concern, 245/5,099 (4.8%) had inadequate image acquisition, 51/5,099 (1.0%) had inadequate image interpretation, and 210/5,099 (4.1%) had images that were either not labeled or labeled inappropriately. Less than 4% (166/5,099) did not have any interpretation associated with the study. Therefore, these could not be assessed for image interpretation. Less than 0.01% had more than one quality concern (32/5,099).

## DISCUSSION

We present the first objective study to quantify POCUS studies performed by community EPs. We found that core studies are performed most often, and there is a higher propensity for these studies to be performed by recent residency graduates.

Many studies have used survey methods to assess exam type without retrospective data to demonstrate actual utilization. For example, a recent Canadian study surveyed EPs and found that FAST, cardiac arrest, and pregnancy were the most commonly reported applications used. In addition, the physician’s age was negatively associated with POCUS use.^10^ A study from 2006 found that respondents who worked in community settings reported using POCUS primarily for FAST, cardiac arrest, and pericardial effusion.^11^ Lastly, a recent survey study assessing non-academic EDs in Arizona found that the most common studies performed at community sites were trauma, cardiac, and line placement.^12^ Our study, using retrospective methods, objectively confirms previous survey data that community EPs perform core exams for life-threatening concerns in community EDs. Additionally, FAST, cardiac, and obstetrical exams were the most common scans performed in our community ED healthcare system, which aligns with previous studies.

Our study also found that less commonly performed exams, such as biliary, joint, DVT, appendix, and testicular ultrasound, were rarely done. There could be many reasons for this, which include an EP’s lack of confidence in performing the exam, difficulty in acquiring correct images, risk of incorrect interpretation, and the amount of time needed to perform a bedside exam. Some of these exams may not routinely be done in EM residency training and, thus, there is limited experience. Additionally, it could be that a physician’s lack of confidence with more complex exams led to opting to obtain radiology-based ultrasound studies. Lastly, it may be that these exams require more time at the bedside, which may prevent community EPs from performing them routinely.

Previous survey studies describe line placement as a more common exam type reported. Given that it is strongly recommended in our system that ultrasound be used for central line placement, we do suspect that there is likely undercounting of its use for line placement. This may be because many of the community EDs do not have the personnel or resources to assist physicians with saving the images during sterile procedures or that it may not have been seen as of critical importance during the procedure and was thus neglected. Therefore, our numbers are likely low as image acquisition into the EHR may limit our ability to truly assess the incidence of POCUS for line placement. However, POCUS for line placement is not a diagnostic modality but rather a procedural aid, and our data does reflect the use of POCUS as a diagnostic modality in the community.

Although we did not perform any statistical comparison analyses to determine statistically significant differences across residency groups in this cohort, the data from our study points to a possible trend suggesting that the greater the number of years from residency graduation, the less likely the EP was to perform POCUS per 100 patients. We suspect physicians who graduated without formal POCUS training during residency are not as likely to pick up this new skill and use it at the bedside. While there is sparse data linking EM residency graduation with the utilization of POCUS in practice, there are smaller studies that demonstrate POCUS training during residency positively impacts clinical utilization within one year after a dedicated course.^13^,^14^

Our study attempted to examine all community EPs in our healthcare system and their POCUS utilization. Objective study numbers performed per 100 patients suggest a trend toward an overall decrease in POCUS utilization with greater number of years from residency graduation. The data may be useful to institutions developing POCUS curricula, training, and credentialing programs for practicing EPs as the data suggests that this population will likely not use POCUS as frequently as EM residency graduates who were trained in POCUS.

One area that deserves discussion from our dataset was the pre-2000 residency graduate group. Our data found an uptick in the group who graduated prior to 2000. To better understand this outlier in the dataset, we performed a detailed analysis, which revealed that two EPs in this group performed 85% of the POCUS exams. These two physicians work at Level 2 trauma centers, which may explain their high utilization of POCUS. When these two physicians were excluded from the pre-2000 cohort, the total number of exams performed dropped from 1,072 to 172 with a group mean of 0.26 POCUS exams per 100 patients. This likely is more representative of this EP group who graduated prior to 2000 and is consistent with the 2000–2004 cohort. However, it raises the question of whether older residency graduates may develop an affinity for POCUS in the proper clinical setting and whether universal POCUS credentialing of EPs is beneficial. Additionally, another question that occurs is whether it is beneficial to train modalities most used in the clinical environment in which the physician practices or whether training should consist of all ACEP-recommended studies.

Finally, quality is a metric that is of utmost importance when performing POCUS exams. Our study demonstrates that POCUS in the community ED produces high-quality, reliable POCUS studies. After a comprehensive credentialing program with imaging compendium recommendations and a standardized reporting system, most exams performed met basic quality metrics for image acquisition, image interpretation, and labeling. This demonstrates that quality studies with compliance can be achieved in the community ED when a comprehensive credentialing program with standardization of POCUS is implemented. This program could be helpful for other specialties or for community hospitals with new or novice scanners trying to implement guidelines and best practices with regard to POCUS.

Overall, the findings from our community assessment are that core POCUS exams are most commonly performed with few quality issues. Hospital systems or new POCUS programs may benefit from focusing on core training, solid competency assessment programs, best practices for privileging and credentialing, and standardizing workflow to ensure high-quality outcomes. Further studies will need to continue to assess the use of POCUS in community EDs to further examine patient outcomes.

## LIMITATIONS

One potential limitations of our study is that the data includes only studies ordered through the EHR with images that were saved and documented within the system and had billing notes completed. It is possible that other studies, known as “phantom scans,” could have been performed at the bedside without image acquisition into the EHR with appropriate documentation. We had no way of assessing the number of “phantom scans” using our current EHR, but we acknowledge that this could be a potential confounder to our study. Along this theme, we believe that most of our community EPs did not save images for central line placement and, thus, there is no data on their performance. However, the healthcare system recommends POCUS as best practice for central line placement.

Another limitation was that a small group of EPs practiced at both academic and community sites within our system. While the study only included their community POCUS exams, these physicians may be more apt to use POCUS due to their exposure at the academic site where residents train in POCUS and perform frequent exams. This may skew this physician group’s POCUS numbers in the community, and we could not account for these few physicians in our study group. In terms of additional limitations related to the physician group, we were unable to obtain retrospective information about how many studies or how frequently the physician group was scanning from the time of their residency graduation to the beginning of the study or continued competency.

Lastly, another variable that could have affected the use of POCUS in community EDs was the availability of 24-hour, radiology-performed and interpreted ultrasound studies. Many of our sites had a 24-hour on-site radiology ultrasound, while others had some limited on-call access during overnight hours. Unfortunately, there was no reliable way to tell when radiology-based ultrasound was unavailable. Possible reasons for community EPs’ limited use of POCUS may be the heavier workload, absence of trainees, and ease of access to radiology-based ultrasound exams, especially when requested by consulting services.

## CONCLUSION

The use of point-of-care ultrasound in community EDs demonstrates a heavy focus on the core exams in EM practice with increased utilization by recent residency graduates. Most studies had no quality issues with image interpretation, image acquisition, or labeling as defined by the guidelines within our privileging program. Our study demonstrates that POCUS can be reliably performed in community EDs and that as our workforce continues to shift toward more recent residency graduates, the use of POCUS in the community EDs will continue to grow. Building standardized infrastructure in community sites to allow for further use of POCUS may be advantageous for healthcare systems.

## Supplementary Information



## Figures and Tables

**Table 1:** Point-of-care ultrasound privileges.

Credentialing tier	Applications	Number of exams required
**Basic ultrasound** (all exam types required for completion)	**General applications:** focused assessment with sonography in trauma (FAST); ultrasound-guided venous access placement; abdominal aorta aneurysm	**FAST: 25 exams** **AAA: 25 exams** **Central Line: 10 exams**
**Intermediate ultrasound** (all exam types required for completion)	**General applications:** pregnancy; echo; biliary; urinary tract; DVT; thoracic; soft tissue/ musculoskeletal; ocular; and procedural guidance	**150 total exams (90 exams if completed Basic Credentialing Tier)**
**Advanced ultrasound** (ability to credential for individual exam types in this category)	Adnexal pathology	**25 exams per exam type**
	Advanced echo	
	Appendicitis	
	Bowel (including intussception)	
	Diverticulitis	
	Pyloric stenosis	
	Small bowel obstruction	
	Testicular	
	Transcranial Doppler	
	Transesophageal echo	
**Requirements for point-of-care ultrasound study**		
Adequate image acquisition		
Adequate image interpretation		
Appropriate labeling of each image		

*AAA*, abdominal aortic aneurysm; *DVT*, deep vein thrombosis; *POCUS*, point-of-care ultrasound.

**Table 2:** Frequency of point-of-care ultrasound exam types in community hospital emergency departments.

Exam category	POCUS exams	% of Total
Cardiac	1,222	24.0%
FAST	1,109	21.7%
Ob	826	16.2%
Skin	491	9.6%
Lung	356	7.0%
Aorta	181	3.5%
Ocular	179	3.5%
Kidney	127	2.5%
Bladder	123	2.4%
Biliary	118	2.3%
E-FAST	113	2.2%
Abdomen other	110	2.2%
Other	59	1.2%
Joint	45	0.9%
DVT	25	0.5%
Appendix	7	0.1%
Procedural ultrasound	7	0.1%
Scrotum	1	0.0%
**GRAND TOTAL**	**5,099**	**100.0%**

*POCUS*, point-of-care ultrasound; *OB*, obstetrics; *FAST*, focused assessment with sonography in trauma*; E-FAST*, extended focused assessment with sonography in trauma; *DVT*, deep vein thrombosis.

**Table 3:** Physician residency graduation year in relation to point-of-care ultrasound exams performed per 100 patients.

Residency group (Yr)	Physician count (N)	Patients (N)	POCUS exams (N)	POCUS exams per 100 patients		

				Group rate	Group mean	Group median
Pre-2000	36	96,556	1,062	1.10	0.78	0.11
2000–2004	21	58,913	127	0.22	0.21	0.03
2005–2009	27	68,674	906	1.32	0.83	0.19
2010–2014	36	120,098	1,019	0.85	0.80	0.33
2015–2019	43	147,202	1,941	1.32	1.30	0.74
2020+	7	2,776	44	1.59	2.03	0.77
**Grand total**	**170**	**494,219**	**5,099**	**1.03**	**0.91**	**0.31**

*POCUS*, point-of-care ultrasound; *Yr*, year.

**Table 4:** Quality metrics for point-of-care ultrasound performed.

		Number of studies (N)
**No quality issues**		4,395
**Quality issue**	Image acquisition (IA)	245
	Image interpretation (II)	51
	Image labeling	210
	No interpretation	166
>**1 Quality issue**	IA + no label	16
	II + no label	7
	IA, II, and no label	9
**Total**		5,099
