# Supplementary material for: A Real-World Experience: Retrospective Review of Point-of-Care Ultrasound Utilization and Quality in Community Emergency Departments
Source: West J Emerg Med. 2023 Jun 30;24(4):685–92. doi: 10.5811/westjem.58965 (PMC10393449; doi:10.5811/westjem.58965)
Supplement: Supplementary file 1 [file wjem-24-685-s001.docx]

# Imaging Compendium

- This is a *suggested* guide for POCUS images for the most common POCUS exams.
- It is highly recommend saving a minimum of 2 views for each study type.

# FAST

1. RUQ (right upper quadrant) – **still** or video
2. LUQ (left upper quadrant) – **still** or video
3. BL TRV, SAG – **still** or video
4. SX (subxiphoid cardiac) – **video**

# Cardiac

1. PSLA (parasternal long axis) - video
2. PSSA (parasternal short axis) - video
3. A4 (Apical 4) - video
4. SX (subxiphoid cardiac) – video

# Chest (lung) – Pneumothorax, Effusion, Infection

1. Lateral Mid axillary line R/L - video
2. Anterior R/L - video
3. Posterior R/L - video

*Each zone can be split into two lung fields

# RUQ

1. Sag GB - **still** or video
2. Trv GB - **still** or video
3. GB Wall measurement – still
4. CBD measurement with color demonstrating appropriate structure - still

# Genitourinary (Kidney+Bladder)

1. R KID TRV (**video**, scan through kidney)
2. R KID SAG (**video**, scan through kidney)
3. L KID TRV (**video**, scan through kidney)
4. L KID SAG (**video**, scan through kidney)
5. BL TRV - **still**

# Bladder

1. 3 measurements of bladder (2 in TRV, 1 in SAG)

# Aorta (positive for > 3cm)

# 5 views is recommended but must obtain a minimum of 3 views

1. Aorta Prox - still
2. Aorta Distal – still
3. Aorta Sagital – still

*1 image must have measurement of Aorta from Outer wall to Outer Wall in the Anterior to posterior orientation.*

5 views

1. Aorta Prox – still
2. Aorta Mid - still
3. Aorta Distal – still
4. Aorta Bifurcation – still (positive for >1.5cm)
5. Aorta Sagital – still

# Ocular

1. R EYE TRV – **video**
2. R EYE SAG– **video**
3. (optional) Contralateral eye– still or **video**

# Pregnancy

1. UT TRV – **video** (sweeping fundus to cervix showing no free fluid)
2. UT SAG – **video** (sweeping right to left showing no free fluid)
3. Image of IUP – still or **video** (Video clip zoomed in area showing the fetal pole with FHT (don’t “sweep”))
4. FHT calculation – **still** (M-mode)

# Soft Tissue Abscess/Cellulitis/Foreign Body

1. Body location TRV - still or **video**
2. Body location SAG - still or **video**

# DVT

1. Prox femoral (at junction of saphenous) without compression - still or **video**
2. Prox femoral (at junction of saphenous) with compression - **video**
3. Femoral Bifurcation without compression - still or **video**
4. Femoral Bifurcation with compression - **video**
5. Popliteal femoral without compression - still or **video**
6. Popliteal femoral with compression - **video**

# Procedures

1. Still Image or video image of targeted area for needle - STATIC
2. Video image of needle entering space (optional) - DYNAMIC
